# Comparison of Vascular Invasion With Lymph Node Metastasis as a Prognostic Factor in Stage I-III Colon Cancer: An Observational Cohort Study

**DOI:** 10.3389/fsurg.2021.773019

**Published:** 2021-11-10

**Authors:** Jung Hoon Bae, Ji Hoon Kim, Bong-Hyeon Kye, Abdullah Al-Sawat, Chul Seung Lee, Seung-Rim Han, In Kyu Lee, Sung Hak Lee, Yoon Suk Lee

**Affiliations:** ^1^Division of Colorectal Surgery, Department of Surgery, Seoul St. Mary's Hospital, College of Medicine, The Catholic University of Korea, Seoul, South Korea; ^2^Division of Colorectal Surgery, Department of Surgery, Incheon St. Mary's Hospital, College of Medicine, The Catholic University of Korea, Incheon, South Korea; ^3^Division of Colorectal Surgery, Department of Surgery, St. Vincent's Hospital, College of Medicine, The Catholic University of Korea, Suwon-Si, South Korea; ^4^Department of Surgery, College of Medicine, Taif University, Taif, Saudi Arabia; ^5^Department of Hospital Pathology, Seoul St. Mary's Hospital, College of Medicine, The Catholic University of Korea, Seoul, South Korea

**Keywords:** colonic neoplasms, vascular invasion, lymph node metastasis, prognosis, recurrence

## Abstract

**Purpose:** This study aimed to evaluate the prognostic impact of vascular invasion (VI) in comparison with that of lymph node metastasis (LNM) in non-metastatic colon cancer.

**Methods:** Patients who underwent curative surgery for stage I-III colon cancer were divided into four groups depending on the status of VI and LNM (Group I: VI-/LNM-; Group II: VI+/LNM-; Group III: VI-/LNM+; Group IV: VI+/LNM+). Group III was subdivided according to the nodal (N) stage (Group IIIA: VI-/N1; Group IIIB: VI-/N2). Oncological outcomes were compared between Groups II and III.

**Results:** In total, 793 non-metastatic colon cancer patients were included. Group II [hazard ratio (HR) 2.34, 1.01–5.41] and Group III (HR 1.91, 1.26–2.89) were independently associated with poor disease-free survival (DFS). The 5-year DFS rates were comparable in Groups II (71.6%) and III (72.5%) (*P* = 0.637). When Group III was subdivided into Groups IIIA and IIIB, DFS deteriorated in the following order: Groups IIIA, II, and IIIB. The 5-year DFS rates were 79.7, 71.6, and 61.4% in Groups IIIA, II, and IIIB, respectively. Group II had a tendency toward early recurrence. The 1- and 2-year DFS rates were 76.3 and 71.6% in Group II and 88.3 and 79.8% in Group III, respectively (*P* = 0.067 and 0.247). All recurrences in Group II were distant metastases.

**Conclusion:** VI is a prognostic factor as significant as LNM and may be a stronger prognostic factor than N1 stage in non-metastatic colon cancer. Furthermore, a potential association was observed between VI and recurrence patterns, such as early recurrence and distant metastasis.

## Introduction

Colon cancer is one of the most common cancers worldwide (1). Standard treatment for non-metastatic colon cancer is curative resection, followed by adjuvant chemotherapy, in selected patients (2, 3). The survival benefit of adjuvant chemotherapy in patients with conventional tumor-node-metastasis (TNM) stage III has been well established (2, 3). Most guidelines recommend adjuvant chemotherapy after curative resection for stage III colon cancer with lymph node metastasis (LNM). In contrast, for stage II colon cancer without LNM, even with high-risk factors, adjuvant chemotherapy is considered an optional treatment modality after curative surgery (2, 3). Generally, high-risk features in colon cancer are as follows: T4 tumors, poorly differentiated tumors, positive margin involvement, <12 lymph nodes (LNs) examined, obstruction, perforation, perineural invasion, lymphatic invasion, and vascular invasion (VI) (2, 3).

Distant metastasis occurs through vascular and lymphatic channels in colon cancer (4–9). However, the conventional TNM staging system categorizes non-metastatic colon cancer into stages I-II and stage III depending only on the status of LNM within the lymphatic pathway (10, 11). The staging system is considered a strong predictor of long-term oncological outcomes.

However, the staging system does not include factors associated with the vascular system, which is a main metastatic pathway. Hence, the impact of vascular metastasis on oncological outcomes has been underestimated compared to that of lymphatic metastasis in the staging system. However, as the vascular system does not have a gateway like LN, we hypothesized that the tumor cells would spread through the vascular channel more aggressively and faster than that through the lymphatic system. In this respect, VI is an important risk factor for distant metastasis through the vascular system, and VI has already been reported in several studies as a significant prognostic factor for colon cancer (12–15). However, there is a lack of literature that directly compares the prognostic effects of VI and LNM. Thus, this study aimed to evaluate the prognostic impact of VI compared to that of LNM in stage I-III colon cancer.

## Materials and Methods

### Patients and Data Collection

**Figure 1:**
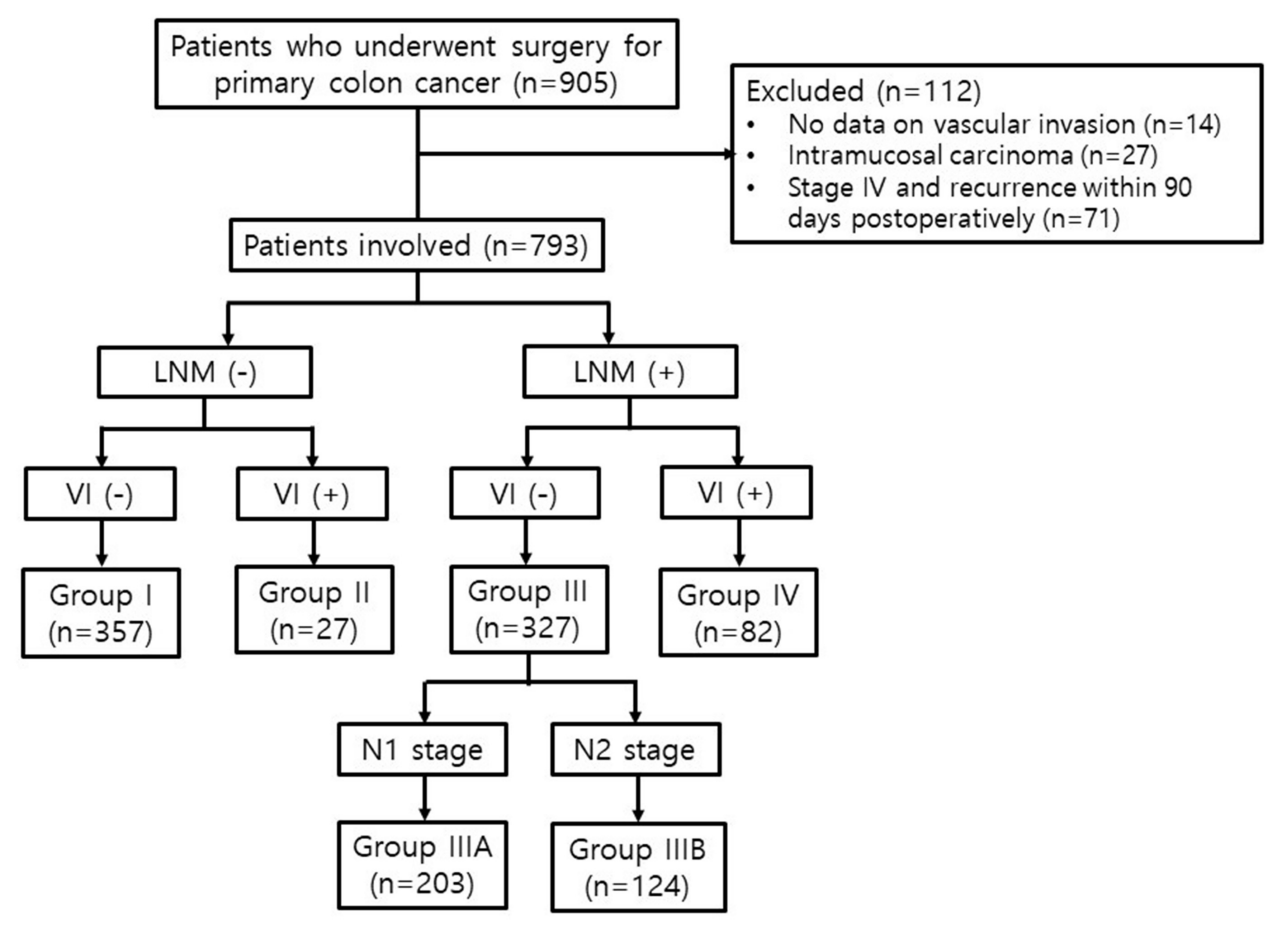
Study flow chart. VI, vascular invasion; LNM, lymph node metastasis.

Patients who underwent curative surgery for primary colon carcinoma between March 2004 and December 2015 at Incheon St. Mary's Hospital were consecutively included in this study (*n* = 905). Patients without data on VI (*n* = 14), those with intramucosal carcinoma (*n* = 27), and those who had a synchronous malignancy at the time of diagnosis or recurrence within 90 days postoperatively (*n* = 71) were excluded. Finally, 793 patients with stage I-III colon cancer were enrolled (Figure 1). This study was conducted by retrospectively reviewing the data, and the follow-up was completed in August 2019.

Patients demographics, clinicopathological characteristics, recurrence, and survival data were collected from the hospital's colon cancer patient registry. Patients with comorbidities were classified according to the American Society of Anesthesiologists (ASA) score. Right-sided colon was defined as cecum, ascending colon, hepatic flexure colon, and transverse colon. Left-sided colon was defined as splenic flexure colon, descending colon, sigmoid colon, and recto-sigmoid colon above the peritoneal reflection.

**Figure 2:**
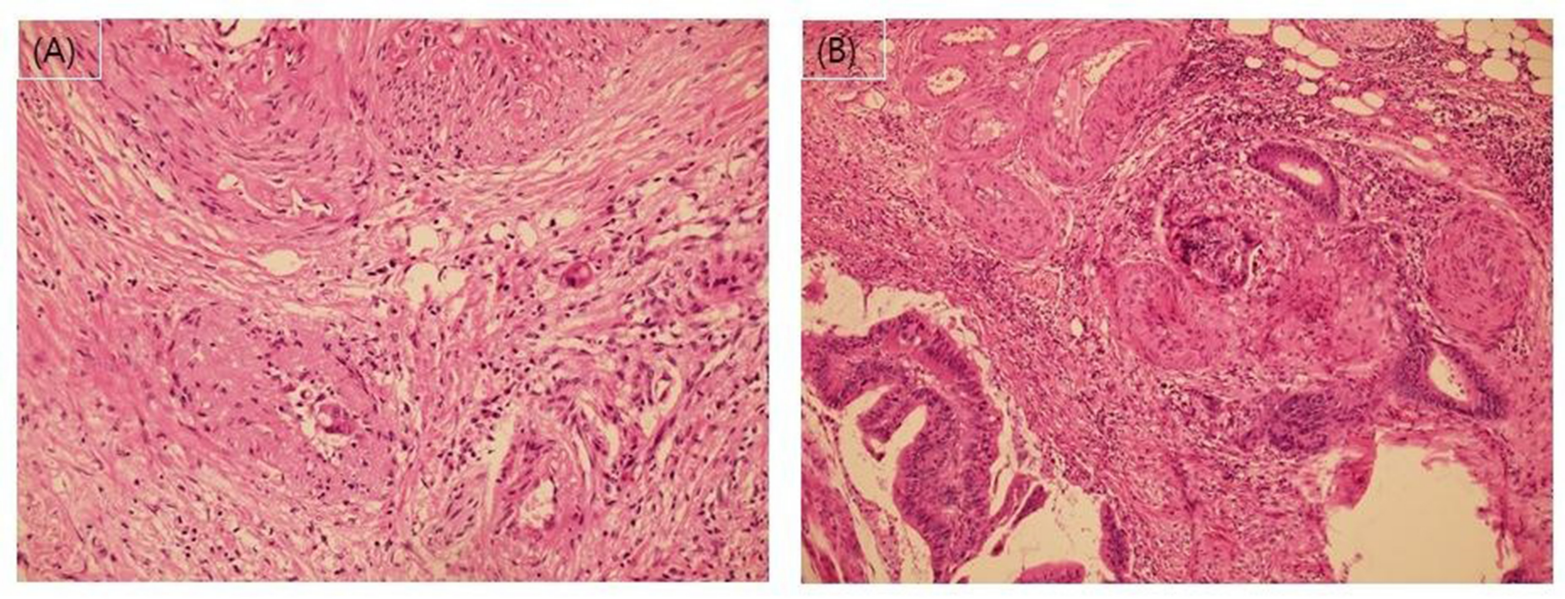
**(A)** and **(B)** Microphotographs of vascular invasion using hematoxylin and eosin staining.

Pathological stage was classified according to the Eighth American Joint Cancer Committee TNM classification system (10, 11). In addition to VI, we recorded lymphatic invasion, perineural invasion, number of examined LNs, and histological grade as high-risk features. A favorable histological grade was defined as well or moderately differentiated adenocarcinoma. A poor histological grade was defined as poorly differentiated adenocarcinoma, mucinous carcinoma, or signet ring cell carcinoma. The presence of lymphovascular invasion was assessed for through hematoxylin and eosin (H&E)-staining. According to the current pathology practice guidelines (16), when the tumor cells involve small vessels with an unequivocal endothelial lining, such as lymphatics, capillaries, and postcapillary venules, it was considered lymphatic (small vessel) invasion. In contrast, when carcinoma was present in vessels with an identifiable thick smooth muscle layer or elastic lamina, this was considered vascular (large vessel) invasion (Figure 2).

This study was approved by the Institutional Review Board of the Ethics Committee of the College of Medicine, The Catholic University of Korea (OC19RESI0035). An informed consent statement was obtained from all patients, and the procedures were carried out in accordance with the ethical standards of the committee responsible for human experimentation and with the guidelines of the Helsinki Declaration, 1975, as revised in 1983. All patient records were anonymized and deidentified before the analysis.

### Study Design

The patients were divided into four groups depending on the status of VI and LNM, regardless of the TNM stage. Group I included patients without VI and LNM (VI-/LNM-); Group II, those with VI and without LNM (VI+/LNM-); Group III, those without VI and with LNM (VI-/LNM+); and Group IV, those with both VI and LNM (VI+/LNM+). Subsequently, we subdivided Group III into Groups IIIA and IIIB according to the N stage. Group IIIA included patients without VI and with N1 stage (metastasis in 1–3 regional LNs), and Group IIIB, those without VI and with N2 stage (metastasis in four or more regional LNs) (Figure 1).

### Follow-Up Schedule and Clinical Outcomes

Surveillance was performed every 3–6 months until 2 years postoperatively and then every 6–12 months until 5 years postoperatively. Tumor markers including carcinoembryonic antigen and carbohydrate antigen 19-9, abdominopelvic computed tomography (CT), and chest CT were performed according to the surveillance schedule. Adjuvant chemotherapy was recommended for patients with stage III and for those with stage II who had at least one high-risk feature. The decision to perform chemotherapy was made taking into consideration the patient's performance status and consent.

The primary outcome was disease-free survival (DFS), which was defined as the interval from the date of surgery until the date of disease recurrence detection by radiological or pathological examination or, in case of no recurrence, until the date of last follow-up. Overall survival (OS) was defined as the interval from the date of surgery until the date of death or last follow-up.

### Statistical Analyses

The categorical variables of the groups were compared using the chi-square or Fisher's exact test. DFS and OS rates were calculated using the Kaplan-Meier method, and survival curves were compared among the groups using the log-rank test. The univariate prognostic significance of variables was determined using the Cox proportional hazard model. Variables significantly related to survival rate in the univariate analysis were subsequently included in the multivariate analysis employing the Cox multiple regression model. All statistical analyses were performed using SPSS software for Windows (version 25.0; IBM Corp., Armonk, NY, USA). Two-tailed *P*-values of < 0.05 were considered statistically significant.

## Results

### Baseline Characteristics Related to VI

In total, 793 patients with stage I-III colon cancer were included in the study. Median follow-up duration was 48 months (interquartile range 29–65). The mean age of the patients was 63.6 ± 12.5 years, the male-to-female ratio was 1.18:1, and 131 patients (16.5%) had an ASA score ≥3. Tumors were localized to the right-sided colon in 304 (38.3%) and the left-sided colon in 489 patients (61.7%). The operation was performed by the laparoscopic approach in 738 (93.1%) and the conventional approach in 55 (6.9%) patients. Adjuvant chemotherapy was administered to 575 (72.5%), and recurrence was observed in 153 patients (19.3%).

VI was observed in 109 patients (13.7%). The patients' clinicopathological characteristics were compared according to the status of VI (Table 1). Patients with VI demonstrated significantly higher rates of lymphatic invasion (89.0 vs. 38.7%; *P* <0.001), perineural invasion (60.6 vs. 32.9%; *P* <0.001), poor histological grade (20.2 vs. 7.9%; *P* <0.001), T4 tumor (37.6 vs. 15.5%; *P* <0.001), and LNM (75.2 vs. 47.8%; *P* <0.001) than those without VI. Adjuvant chemotherapy was performed more in patients with VI than in those without VI (82.6 vs. 70.9%; *P* = 0.011). The recurrence rate was higher in patients with VI than in those without VI (29.4 vs. 17.7%; *P* = 0.004). However, local recurrence rates were not different (2.8 vs. 2.0%; *P* = 0.718).

**Table 1:** Patient's clinico-pathological characteristics according to the presence of vascular invasion (VI).

**Variables**	**Total patients** **(*N* = 793)**	**No VI (%)** **(*N* = 684, 86.3%)**	**VI (%)** **(*N* = 109, 13.7%)**	***P***
Age > 65 years	397 (50.1)	341 (49.9)	56 (51.4)	0.768
Sex, female	363 (45.8)	320 (46.8)	43 (39.4)	0.153
ASA score, ≥3	131 (16.5)	111 (16.2)	20 (18.3)	0.580
Tumor location				0.220
Right-sided	304 (38.3)	268 (39.2)	36 (33.0)	
Left-sided	489 (61.7)	416 (60.8)	73 (67.0)	
Surgical approach				0.858
Laparoscopic	738 (93.1)	637 (93.1)	101 (92.7)	
Conventional	55 (6.9)	47 (6.9)	8 (7.3)	
Combined resection, yes	109 (13.7)	96 (14.0)	13 (11.9)	0.553
T4 tumor	147 (18.5)	106 (15.5)	41 (37.6)	<0.001
LN metastasis, yes	409 (51.6)	327 (47.8)	82 (75.2)	<0.001
Stage				<0.001
I	54 (6.8)	52 (7.6)	2 (1.8)	
II	330 (41.6)	305 (44.6)	25 (22.9)	
III	409 (51.6)	327 (47.8)	82 (75.2)	
LN harvest, <12	71 (9.0)	61 (8.9)	10 (9.2)	0.931
Histological grade, poor	76 (9.6)	54 (7.9)	22 (20.2)	<0.001
Lymphatic invasion, yes	362 (45.6)	265 (38.7)	97 (89.0)	<0.001
Perineural invasion, yes	291 (36.7)	225 (32.9)	66 (60.6)	<0.001
Adjuvant chemotherapy, yes	575 (72.5)	485 (70.9)	90 (82.6)	0.011
Recurrence, yes	153 (19.3)	121 (17.7)	32 (29.4)	0.004
Local recurrence, yes	17 (2.1)	14 (2.0)	3 (2.8)	0.718

*VI, vascular invasion; ASA, American Society of Anesthesiologist; T, tumor; LN, lymph node*.

*Proportion are presented for categorical data (%)*.

### Baseline Characteristics According to the Groups

Of the 793 patients, 357 (45.0%) were included in Group I, 27 (3.4%) in Group II, 327 (41.2%) in Group III, and 82 (10.3%) in Group IV. The patients' clinicopathological characteristics are listed according to their respective groups in Table 2. Group IV (VI+/LNM+) had the highest rates of T4 tumor (*P* < 0.001), poor histological grade (*P* < 0.001), lymphatic invasion (*P* < 0.001), and perineural invasion (*P* < 0.001) among the groups. The recurrence rate was the highest in Group IV (30.5%) and the lowest in Group I (11.5%) (*P* < 0.001). There was no difference in local recurrence rates among the Groups (*P* = 0.388).

**Table 2:** Patient's clinico-pathological characteristics according to the groups.

**Variables**	**Group I** **VI-, LNM-** **(*N* = 357)**	**Group II** **VI+, LNM-** **(*N* = 27)**	**Group III** **VI-, LNM+** **(*N* = 327)**	**Group IV** **VI+, LNM+** **(*N* = 82)**	***P* ^**a**^**	***P* ^**b**^**
Age > 65 years	180 (50.4)	18 (66.7)	161 (49.2)	38 (46.3)	0.316	0.082
Sex, female	152 (42.6)	8 (29.6)	168 (51.4)	35 (42.7)	0.033	0.030
ASA score, ≥ 3	56 (15.7)	6 (22.2)	55 (16.8)	14 (17.1)	0.836	0.435
Tumor location					0.518	0.174
Right-sided	140 (39.2)	7 (25.9)	128 (39.1)	29 (35.4)		
Left-sided	217 (60.8)	20 (74.1)	199 (60.9)	53 (64.6)		
Surgical approach					0.422	1.000
Laparoscopic	338 (94.7)	25 (92.6)	299 (91.4)	76 (92.7)		
Conventional	19 (5.3)	2 (7.4)	28 (8.6)	6 (7.3)		
Combined resection, yes	45 (12.6)	2 (7.4)	51 (15.6)	11 (13.4)	0.520	0.399
T4 tumor	30 (8.4)	6 (22.2)	76 (23.2)	35 (42.7)	<0.001	0.904
Stage					<0.001	<0.001
I	52 (14.6)	2 (7.4)	0	0		
II	305 (85.4)	25 (92.6)	0	0		
III	0	0	327 (100)	82 (100)		
LN harvest, <12	39 (10.9)	4 (14.8)	22 (6.7)	6 (7.3)	0.165	0.125
Histological grade, poor	18 (5.0)	2 (7.4)	36 (11.0)	20 (24.4)	<0.001	0.753
Lymphatic invasion, yes	67 (18.8)	21 (77.8)	198 (60.6)	76 (92.7)	<0.001	0.077
Perineural invasion, yes	93 (26.1)	12 (44.4)	132 (40.4)	54 (65.9)	<0.001	0.678
Adjuvant systemic chemotherapy, yes	218 (61.1)	21 (77.8)	267 (81.7)	69 (84.1)	<0.001	0.619
Recurrence, yes	41 (11.5)	7 (25.9)	80 (24.5)	25 (30.5)	<0.001	0.865
Local recurrence, yes	5 (1.4)	0 (0.0)	9 (2.8)	3 (3.7)	0.388	1.000

*VI, vascular invasion; LNM, lymph node metastasis; ASA, American Society of Anesthesiologist; T, tumor; LN, lymph node*.

a *P-value comparing all groups*.

b *P-value comparing only group II (VI+/LNM-) and group III (VI-/LNM+)*.

*Proportion are presented for categorical data (%)*.

No significant differences were observed between Groups II (VI+/LNM-) and III (VI-/LNM+) in baseline characteristics, except for the sex ratio; patients in Group II were predominantly male (*P* = 0.030). Adjuvant chemotherapy was administered in 77.8 and 81.7% of patients in Groups II and III, respectively (*P* = 0.619). Recurrence rates were 25.9 and 24.5% in Groups II and III, respectively (*P* = 0.865).

### Survival Outcomes

The outcomes of the univariate and multivariate analyses to identify significant prognostic factors for DFS and OS are shown in Tables 3, 4, respectively. The univariate analysis showed that both VI [hazard ratio (HR) 1.98; 95% confidence interval (CI) 1.34–2.92] and LNM (HR 2.26; 95% CI 1.60–3.17) were significant prognostic factors for poor DFS. Multivariate analysis revealed that Group II (HR 2.34; 95% CI 1.01–5.41), Group III (HR 1.91; 95% CI 1.26–2.89), Group IV (HR 2.34; 95% CI 1.33–4.14), poor histological grade (HR 1.66; 95% CI 1.07–2.59), and T4 tumors (HR 2.08; 95% CI 1.44–3.01) were independently associated with poor DFS. Lymphatic invasion and perineural invasion were not detected as significant prognostic factors for DFS.

**Table 3:** Disease-free survival in stage I-III colon cancer patients.

	**Univariate analysis**		**Multivariate analysis**	
**Variables**	**HR (95% CI)**	***P***	**HR (95% CI)**	***P***
Age ≥ 65	1.29 (0.94–1.77)	0.119		
Sex, female	0.90 (0.65–1.24)	0.512		
ASA score, ≥ 3	1.24 (0.82–1.89)	0.304		
Tumor location, left-sided	0.98 (0.71–1.36)	0.912		
T4 tumor	2.75 (1.96–3.85)	<0.001	2.08 (1.44–3.01)	<0.001
LN metastasis, yes	2.26 (1.60–3.17)	<0.001		
LN harvest, <12	1.01 (0.57–1.79)	0.970		
Histological grade, poor	2.05 (1.32–3.16)	0.001	1.66 (1.07–2.59)	0.024
VI, yes	1.98 (1.34–2.92)	0.001		
Lymphatic invasion, yes	1.89 (1.37–2.61)	<0.001	1.04 (0.70–1.55)	0.847
Perineural invasion, yes	1.52 (1.11–2.09)	0.010	1.09 (0.77–1.56)	0.617
Adjuvant systemic chemotherapy, yes	0.84(0.58–1.21)	0.340		
**Group**				
I (VI-, LNM-)	Reference		Reference	
II (VI+, LNM-)	2.78 (1.25–6.20)	0.012	2.34 (1.01–5.41)	0.048
III (VI-, LNM+)	2.31 (1.59–3.37)	<0.001	1.91 (1.26–2.89)	0.002
IV (VI+, LNM+)	3.29 (2.00–5.42)	<0.001	2.34 (1.33–4.14)	0.003

*HR, hazard ratio; CI, confidence interval; ASA, American Society of Anesthesiologist; T, tumor; LN, lymph node; VI, vascular invasion; LNM, lymph node metastasis*.

**Table 4:** Overall survival in stage I-III colon cancer patients.

	**Univariate analysis**		**Multivariate analysis**	
**Variables**	**HR (95% CI)**	***P***	**HR (95% CI)**	***P***
Age ≥ 65	1.96 (1.29–3.00)	0.002	1.53 (0.97–2.39)	0.066
Sex, female	0.67 (0.43–1.02)	0.062		
ASA score, ≥ 3	2.07 (1.30–3.30)	0.002	1.64 (1.01–2.66)	0.044
Tumor location, left-sided	0.94 (0.61–1.43)	0.754		
T4 tumor	2.59 (1.67–4.01)	<0.001	1.98 (1.21–3.23)	0.007
LN metastasis, yes	1.87 (1.22–2.88)	0.004		
LN harvest, <12	1.62 (0.88–2.97)	0.122		
Histological grade, poor	2.54 (1.52–4.26)	<0.001	2.11 (1.24–3.61)	0.006
VI, yes	2.21 (1.37–3.58)	0.001		
Lymphatic invasion, yes	1.79 (1.18–2.72)	0.006	1.21 (0.71–2.06)	0.481
Perineural invasion, yes	1.38 (0.92–2.10)	0.124		
Adjuvant systemic chemotherapy, yes	0.43(0.28–0.65)	<0.001	0.33 (0.20–0.53)	<0.001
**Group**				
I (VI-, LNM-)	Reference		Reference	
II (VI+, LNM-)	2.03 (0.71–5.79)	0.185	1.72 (0.57–5.22)	0.340
III (VI-, LNM+)	1.71 (1.06–2.76)	0.029	1.66 (0.85–3.27)	0.141
IV (VI+, LNM+)	3.26 (1.81–5.90)	<0.001	2.92 (1.40–6.08)	0.004

*HR, hazard ratio; CI, confidence interval; ASA, American Society of Anesthesiologist; T, tumor; LN, lymph node; VI, vascular invasion; LNM, lymph node metastasis*.

Both VI (HR 2.21; 95% CI 1.37–3.58) and LNM (HR 1.87; 95% CI 1.22–2.88) were significant prognostic factors for poor OS in the univariate analysis. In the multivariate analysis, high ASA score (HR 1.64; 95% CI 1.01–2.66), T4 tumor (HR 1.98; 95% CI 1.21–3.23), poor histological grade (HR 2.11; 95% CI 1.24–3.61), and Group IV (HR 2.92; 95% CI 1.40–6.08) were independently associated with poor OS. Adjuvant chemotherapy was an independent favorable prognostic factor for OS (HR 0.33; 95% CI 0.20–0.53).

**Figure 3:**
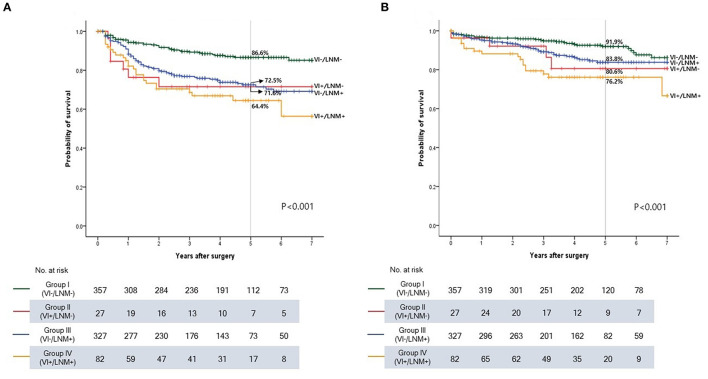
Kaplan-Meier survival curve according to the groups in stage I-III colon cancer. **(A)** Disease-free survival. **(B)** Overall survival. VI, vascular invasion; LNM, lymph node metastasis.

The Kaplan-Meier curves for DFS and OS are shown according to the groups (Figure 3). Patients in Groups II (VI+/LNM-) and III (VI-/LNM+) had poorer prognosis than those in Group I (VI-/LNM-). Group IV (VI+/LNM+) showed the worst prognosis among the groups regarding DFS and OS (*P* < 0.001 and *P* < 0.001). The 5-year DFS rates were 86.6, 71.6, 72.5, and 64.4% in Groups I, II, III, and IV, respectively. The 5-year OS rates were 91.9, 80.6, 83.8, and 76.2% in Groups I, II, III, and IV, respectively.

### Comparison of Oncological Outcomes Between Groups II (VI+/LNM-) and III (VI-/LNM+)

**Figure 4:**
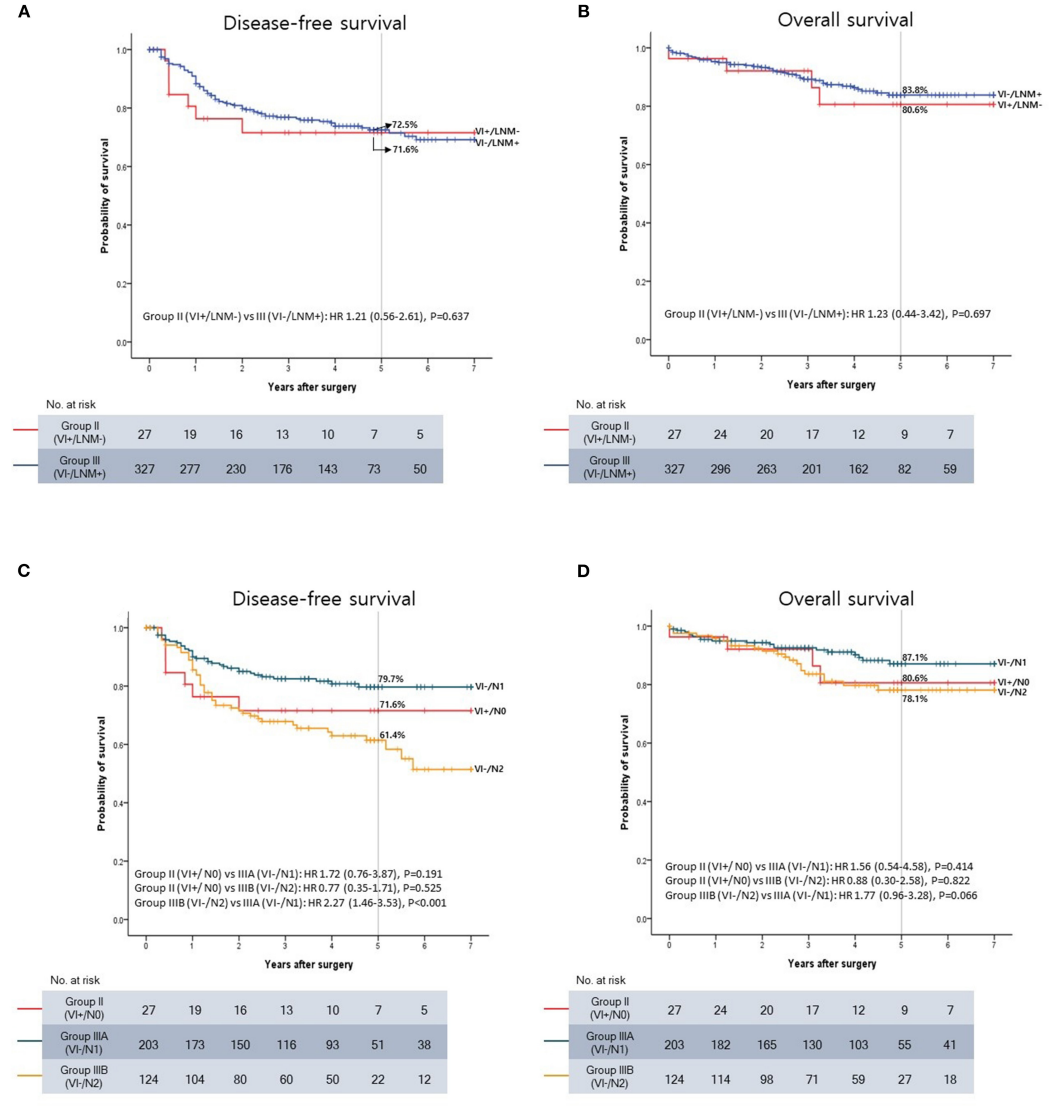
Kaplan-Meier survival curve. **(A)** Disease-free survival (DFS) between VI+/LNM- and VI-/LNM+ patients. **(B)** Overall survival (OS) between VI+/LNM- and VI-/LNM+ patients. **(C)** DFS between VI+/LNM-, VI-/N1, and VI-/N2 patients. **(D)** OS between VI+/LNM-, VI-/N1, and VI-/N2 patients.

A survival analysis including only Groups II and III was performed to directly compare the prognostic impact of VI and LNM. No significant differences in DFS and OS were observed between Groups II and III (*P* = 0.637 and *P* = 0.697) (Figures 4A,B).

However, the 1- and 2-year DFS rates tended to be lower in Group II than in Group III. The 1-year DFS rates were 76.3% in Group II and 88.3% in Group III (*P* = 0.067), and the 2-year DFS rates were 71.6% in Group II and 79.8% in Group III (*P* = 0.247). In contrast, the 5-year DFS rates were almost the same between the two groups (71.6% and 72.5% in Groups II and III, respectively).

Subsequently, Group III was subdivided into Groups IIIA (VI-/N1) and IIIB (VI-/N2) on the basis of the N stage, and the oncological outcomes were compared to those in Group II (VI+/N0). The Kaplan-Meier curve showed that DFS and OS deteriorated in the following order: Groups IIIA, II, and IIIB (Figures 4C,D). The 5-year DFS rates were 79.7, 71.6, and 61.4% in Groups IIIA, II, and IIIB, respectively. The 5-year OS rates were 87.1, 80.6, and 78.1% in Groups IIIA, II, and IIIB, respectively.

### Recurrence Patterns

No differences in recurrence patterns such as distant metastasis, local recurrence, or recurrence in the liver, lung, or peritoneum were observed between patients in Groups II and III (Table 5) with recurrences. However, all recurrences were distant metastasis in Group II. In contrast, local recurrence was observed in nine patients (11.3%) in Group III.

**Table 5:** Recurrence types of recurrent patients in groups II and III.

**Variables**	**Group II** **VI+, LNM-** **(*N* = 7)**	**Group III** **VI-, LNM+** **(*N* = 80)**	***P***
Liver metastasis, yes	3 (42.9)	33 (41.3)	1.000
Lung metastasis, yes	2 (28.6)	26 (32.5)	1.000
Peritoneal metastasis, yes	2 (28.6)	17 (21.3)	0.644
Distant metastasis, yes	7 (100.0)	75 (93.8)	1.000
Local recurrence, yes	0 (0.0)	9 (11.3)	1.000

*VI, vascular invasion; LNM, lymph node metastasis*.

*Proportion are presented for categorical data (%)*.

## Discussion

In colon cancer, the TNM staging system is very simple because LNM is the only criterion for stage III (10). However, overemphasis on LNM in the staging system is controversial. Many studies have reported the poor prognostic impact of high-risk features other than LNM (17–21). One study showed the relationship between the number of high-risk features and prognosis, demonstrating that the 5-year OS rate was <20% in patients with stage II colon cancer with four or more high-risk features (20).

In this study, we compared the prognostic impact of VI, one of the high-risk features, with that of LNM, the criteria for stage III in the TNM staging system. We divided the patients into groups depending on their VI and LNM statuses, and no significant difference between the prognostic impacts of VI and LNM was found. Furthermore, despite no statistical significance, the 5-year DFS rate in VI+/N0 patients (71.6%) was lower than that in VI-/N1 patients (79.7%) and higher than that in VI-/N2 patients (61.4%). These findings suggest that the prognostic impact of VI may be somewhere between those of N1 and N2 in the TNM staging system.

Another interesting point of this study was the recurrence pattern. Seven recurrences occurred in VI+/LNM- patients, and all seven (100%) occurred within 2 years. Of the seven recurrences, six cases (85.7%) occurred within 1 year after surgery, and one case (14.3%) occurred 24 months after surgery. In VI-/LNM+ patients, there were 36 (45.0%) recurrences within 1 year and 61 (76.3%) recurrences within 2 years among the 80 recurrences in total. The 1-year DFS rates were 76.3% in VI+/LNM- patients and 88.3% in VI-/LNM+ patients, and the 2-year DFS rates were 71.6% in VI+/LNM- patients and 79.8% in VI-/LNM+ patients. Additionally, all recurrences in VI+/LNM- patients were distant metastasis. In contrast, 11.3% of the recurrences in VI-/LNM+ patients were local recurrences. Although there was no statistical significance, VI+/LNM- was found to be associated with early recurrence and distant metastasis compared with VI-/LNM+. In this study, the proportion of VI+/LNM- patients who underwent chemotherapy was 77.8%, comparable to the number of VI-/LNM+ patients who underwent chemotherapy (81.7%). Therefore, the postoperative factors affecting oncological outcomes were minimized.

Distant metastasis is the most common cause of death in patients with cancer (22, 23), and it is known to occur through vascular and lymphatic channels (4–9). Lymphatic drainage occurs in the following order: epicolic/paracolic LN, intermediate LN, and apical LN in colon cancer (24), and an association between the location of a regional LNM and disease recurrence has been reported (25, 26). Therefore, complete mesocolic excision (CME) and central vessel ligation (CVL), including the removal of apical LNs, are considered standard procedures in colon cancer surgery. Indeed, many studies have reported that CME and CVL contributed to better survival outcomes (27–29). When surgeons perform adequate CME and CVL procedures, the risk for metastasis through lymphatic channels can be reduced. For example, in stage III colon cancer without apical LNM, theoretically, the possibility of distant metastasis through lymphatic channels is negated by surgery with CME and CVL if there is no skip LNM. In contrast, the vascular system differs from the lymphatic system because it does not have a gateway like LNs. On the basis of these facts, tumor cell dissemination would be more aggressive and faster when tumor cells were transported through the vascular system than through the lymphatic system. In this study, we performed CME and CVL in all patients and demonstrated that VI is not only a poor prognostic factor similar to LNM, but also an indicator of early recurrence.

This study had several limitations. First, as this study was retrospective, inherent and unintentional selection bias cannot be dismissed. However, the selection bias was minimized because the clinicopathological factors, which can affect the prognosis, were not different between VI+/LNM- and VI-/LNM+ patients. Even the proportions of patients who received adjuvant chemotherapy among VI+/LNM- and VI-/LNM+ patients were comparable. Second, the sample size was small. The 1- and 2-year DFS rates of VI+/LNM- patients were lower than those of VI-/LNM+ patients. Furthermore, VI tended to be a stronger prognostic factor than metastasis in 1–3 regional LNs (N1 stage). However, these differences were not statistically significant. Third, we did not include MSI status as a prognostic factor in this study. According to the ESMO guideline, vascular invasion is classified as a minor prognostic parameter for stage II risk assessment (3). The guideline recommends that adjuvant therapy be determined based on the MSI status in intermediate-risk stage II colon cancer. The study center has evaluated the MSI status as a prognostic factor since 2013. Therefore, most of them have no data on MSI status. Since adjuvant chemotherapy was performed for stage II colon cancer with at least one risk factor regardless of MSI status, compared with current ESMO guideline, some of the patients included in this study underwent overtreatment. Finally, the detection rate of VI was low in this study (13.7%). Recent studies have reported detection rates of VI ranging from 19 to 34% (12–14, 17, 18, 20). This difference may be due to the staining method. One study investigated the detection rate of VI in 93 patients with T3 or T4 colorectal cancer and reported that it increased from 15.1% in the original pathological report when using H&E staining to 48.4% when using elastic stain (30). During the study period, we used H&E staining for the detection of VI, which could result in a low VI detection rate. This may be the reason why only a small population belonged to Group II (VI+/LNM-). Therefore, a large-scale multicenter study using elastin staining as a method to detect VI is needed to clarify the results of this study.

In conclusion, we demonstrated that VI is a prognostic factor as significant as LNM and may be a stronger prognostic factor than the N1 stage in non-metastatic colon cancer. Furthermore, the result provided the first insights into a potential association between VI and recurrence patterns, such as early recurrence and distant metastasis. Therefore, this study suggests that adjuvant chemotherapy should be considered in stage II colon cancer with VI. A large-scale multicenter study using an advanced staining method would help to clarify the prognostic impact of VI.

## Data Availability Statement

The original contributions presented in the study are included in the article/supplementary material, further inquiries can be directed to the corresponding author/s.

## Ethics Statement

The studies involving human participants were reviewed and approved by Institutional Review Board of the Ethics Committee of the College of Medicine, the Catholic University of Korea (OC19RESI0035). Written informed consent for participation was not required for this study in accordance with the national legislation and the institutional requirements.

## Author Contributions

YL: conceptualization and writing-review and editing. JK and YL: data curation. JB: formal analysis, software, and writing–original draft. JB, CL, S-RH, JK, and YL: investigation. JB, B-HK, AA-S, SL, and YL: methodology. JB and YL: project administration. JB, JK, and YL: resources. B-HK, IL, and YL: supervision and validation. JB, SL, and YL: visualization.

## Conflict of Interest

The authors declare that the research was conducted in the absence of any commercial or financial relationships that could be construed as a potential conflict of interest.

## Publisher's Note

All claims expressed in this article are solely those of the authors and do not necessarily represent those of their affiliated organizations, or those of the publisher, the editors and the reviewers. Any product that may be evaluated in this article, or claim that may be made by its manufacturer, is not guaranteed or endorsed by the publisher.
